# A new clinical-genomic model to predict 10-year recurrence risk in primary operable breast cancer patients

**DOI:** 10.1038/s41598-020-61535-9

**Published:** 2020-03-17

**Authors:** Tzu-Ting Huang, Lei Lei, Ching-Hsuan Andre Chen, Tzu-Pin Lu, Chung-Wen Jen, Skye Hung-Chun Cheng

**Affiliations:** 1 Department of Research, Koo Foundation, Sun Yat-Sen Cancer Center, Taipei, Taiwan; 2 Department of Breast Medical Oncology, Cancer Hospital of University of Chinese Academy of Sciences (Zhejiang Cancer Hospital), ZheJiang, China; 3 Visiting Scholar, University of Mississippi Medical Center, Jackson, Mississippi USA; 4 Epidemiology and Preventive Medicine, Department of Public Health, National Taiwan University, Taipei, Taiwan; 5 Department of Radiation Oncology, Koo Foundation Sun Yat-Sen Cancer Center, Taipei, Taiwan; 6 Taitung Cancer Center, Department of Radiation Oncology, Taitung Christian Hospital, Taitung, Taiwan

**Keywords:** Breast cancer, Prognostic markers

## Abstract

This study aimed to validate the long-term prognostic value of a new clinical-genomic model, Distant Genetic Model-Clinical Variable Model 6 (DGM-CM6), developed in Asia as a prognostic panel for all subtypes of breast cancer. We included 752 operable stage I–III breast cancer patients representing all subtypes treated from 2005 to 2014 as the validation cohort. The median follow-up was 95.8 months. The low- and high-risk patients classified by DGM-CM6 (RI-DR) had significant differences in 10-year distant recurrence-free interval (DRFI) (94.1% vs. 85.0%, *P* < 0.0001) and relapse-free survival (RFS) (90.0% vs. 80.5%, *P* = 0.0003). External validation using EMTAB-365 dataset showed similar observation (*P* < 0.0001). DGM-CM6 was an independent prognostic factor by multivariate analysis with hazard ratios of 3.1 (1.6–6.0) for RFS (*P* = 0.0009) and 3.8 (1.6–9.0) for DRFI (*P* = 0.0028). Comparing the C-index of DGM-CM6 and PAM50-ROR scores, the former performed better than the latter in predicting long-term DRFI and RFS, especially in N0, ER/PR-positive, and HER2-negative patients.

## Introduction

Breast cancer is the most prevalent and deadly malignant disease among women throughout the world. Despite recent advances in early breast cancer (EBC) management, recurrent events remain inevitable in high-risk populations^1^. A reliable prognostic algorithm combining clinical and genomic information to help determine treatment strategies for EBC patients is urgently needed. The Oncotype DX (ODx) 21-gene recurrence score (RS) is known to be a sound prognostic and predictive assay in node-negative hormonal receptor-positive (HR-positive) and human epidermal growth factor receptor 2 (HER2) negative breast cancer patients. However, its prognostic value in HR-negative patients is unknown. According to the Trial Assigning Individualized Options for Treatment (TAILORx), the RS cutoff at which chemotherapy in patients 50 years of age or younger can safely be avoided is under investigated^2,3^. Women from Asian-Pacific countries experience earlier onset of breast cancer than women from Western countries, nearly 50% suffering from breast cancer under the age of 50^4^. However, they usually have a better survival rate. One study using data from Surveillance, Epidemiology and End Results (SEER) demonstrated that the actuarial risk of death 7 years post-onset for women with stage I breast cancer was lower among Asian women than that of non-Hispanic white women^5^. This difference may be related to biological differences in tumour characteristics between the races. The ODx was mainly developed based on Western populations and may not be fully applicable to Asian ethnic groups; the TAILORx trial included only 151 Asian women out of a total of 11,248 (1.3%) patients^2^. Overestimated prediction for the recurrence risk by ODx in Japanese populations has been reported, with no recurrence in the intermediate-risk group (cutoff 18–30)^6^. Considering the potential impact caused by differences in race and ethnicity, a Korean group developed a multi-gene assay in 2019, which could identify more low-risk patients in the young age group (<50 years) than those identified by ODx^7^. In our group, a 34-gene panel was developed in 2006, which could classify the low- and high-risk groups of local/regional recurrence (LRR) after mastectomy^8^. This multi-gene panel was further refined and validated as an 18-gene classifier (18-GC) with more sensitivity, specificity, and accuracy—not only in predicting LRR but also distant recurrence^9,10^. This 18-GC utilises the genes *BLM*, *TCF3*, *PIM1*, *RCHY1*, *PTI1*, *DDX39*, *BUB1B*, *STIL*, *TPX2*, *CCNB1*, *MMP15*, *CCR1*, *NFATC2IP*, *TRPV6*, *OBSL1*, *C16ORF7*, *DTX2*, *and ENSA*, among which 17 were included in the distant genomic model (DGM).

This clinical model was based on our previous work that identified the most important prognostic factors as the number of axillary lymph nodes involved, age at diagnosis (≤40vs >40 years), prominent lymphovascular invasion (LVI), oestrogen receptor (ER) status, tumour grade, and tumour size (>2 cm)^11,12^. Incorporating both the genomic and clinical data, DGM-CM6 (recurrence index for distant metastasis [RI-DR]) proved to be the most predictive^13^.

In this study, we assessed and validated the prognostic value of DGM-CM6 (RI-DR) in different molecular subtypes of EBC after surgery based on the independent dataset.

## Results

### Validation dataset

A total of 752 patients who had undergone Affymetrix microarray testing and had N0-2 breast cancer were included in the analysis (mastectomy, n = 482; BCS, n = 270). The median follow-up was 86.9 months for patients without adjuvant chemotherapy and 96 months for patients with chemotherapy. Patients without adjuvant chemotherapy were significantly older and had favourable pathological features (T1, HR-positive, HER2-negative, no/focal LVI, and grade I/II) (Supplementary Table S3). The median age of subjects was 49 years (range: 27–88 years), 55.5% (417) were 50 or below and 64.0% (481) were pre-menopausal. Immunohistochemical analysis revealed that 34.6% (260) of subjects were negative for both ER and progesterone receptor (PR) and 34.2% (257) were positive for HER2. Prominent LVI tumours were identified in 22.6% (170) of patients. Adjuvant chemotherapy was used in 89.1% (670) of patients and adjuvant hormone therapy was used in 62.6% (471) of patients. Among mastectomy patients, post-mastectomy radiotherapy was administrated in 65.1% (314/482). Among HER2-positive patients, 37.7% (97/257) received adjuvant trastuzumab **(**Table 1**)**.

**Table 1:** Baseline characteristics of subjects in the internal validation dataset.

Variables	All patients (n = 752)	Low-risk* (n = 232)	High-risk* (n = 520)	P-value
Median follow-up (Months)	95.8 (0.6–169.3)	95.8 (0.6–169.3)	95.8 (0.9–164.5)	0.2525
Menstruation status				<0.0001
Pre-menopausal	481	138 (59.5%)	343 (66.0%)	
Post-menopausal	262	91 (39.2%)	171 (32.9%)	
Unknown	9	3 (1.3%)	6 (1.2%)	
Age				<0.0001
<40	138	25 (10.8%)	113 (21.7%)	
41–50	279	101 (43.5%)	178 (34.2%)	
51–60	223	58 (25.0%)	165 (31.7%)	
>60	112	48 (20.7%)	64 (12.3%)	
T stage				<0.0001
T1	327	152 (65.5%)	175 (33.7%)	
T2	408	76 (32.8%)	332 (63.9%)	
T3	17	4 (1.7%)	13 (2.5%)	
N stage				<0.0001
N0	364	152 (65.5%)	212 (40.8%)	
N1	282	67 (28.9%)	215 (41.4%)	
N2	106	13 (5.6%)	93 (17.9%)	
ER and PR status				<0.0001
Both Negative	260	14 (6.0%)	246 (47.3%)	
ER or PR (+)	492	218 (94.0%)	274 (52.7%)	
HER2 overexpression				<0.0001^*^
Negative	492	198 (85.3%)	294 (56.5%)	
Positive	257	34 (14.7%)	223 (42.9%)	
Indeterminant	3	0 (0.0%)	3 (0.6%)	
LVI				<0.0001
Absent/focal	582	201 (86.6%)	381 (73.3%)	
Prominent	170	31 (13.4%)	139 (26.7%)	
Tumour grade				<0.0001
Grade I	83	74 (31.9%)	9 (1.7%)	
Grade II	238	123 (53.0%)	115 (22.1%)	
Grade III	431	35 (15.1%)	396 (76.2%)	
PMRT or RNI				<0.0001
No	184	78 (33.6%)	106 (20.4%)	
Yes	568	154 (66.4%)	414 (79.6%)	
Adjuvant C/T				<0.0001
No	82	54 (23.3%)	28 (5.4%)	
Yes	670	178 (76.7%)	492 (94.6%)	
Adjuvant H/T				<0.0001
No	281	20 (8.6%)	261 (50.2%)	
Yes	471	212 (91.4%)	259 (49.8%)	
Adjuvant trastuzumab				<0.0001
No	655	220 (94.8%)	435 (83.7%)	
Yes	97	12 (5.2%)	85 (16.4%)	
**IHC subtype**				<0.0001
ER/PR+ HER2−, Gr 1–2	249	171(73.7%)	78 (15.0%)	
ER/PR+ HER2−, Gr 3	89	19 (8.2%)	70 (13.5%)	
ER/PR+ HER2+	152	28 (12.1%)	124 (23.9%)	
ER−, PR−, HER2+	105	6 (2.6%)	99 (19.0%)	
ER−, PR−, HER2−	154	8 (3.5%)	146 (28.1%)	
**PAM50 intrinsic subtypes**				<0.0001
Luminal A	192	148 (63.8%)	44 (8.5%)	
Luminal B	212	43 (18.5%)	169 (32.5%)	
HER2	140	1 (0.4%)	139 (26.7%)	
Basal	144	2 (0.9%)	142 (27.3%)	
Normal	64	38 (16.4%)	26 (5.0%)	

^*^Defined by DGM-CM6: cutoff <33 as low-risk, $\ge$33 as high-risk.

BCS, breast-conserving surgery; C/T, chemotherapy; H/T, hormonal therapy; LVI, lymphovascular invasion; MRM, modified radical mastectomy; PMRT, post-mastectomy radiotherapy; RNI, regional node irradiation.

**Figure 1:**
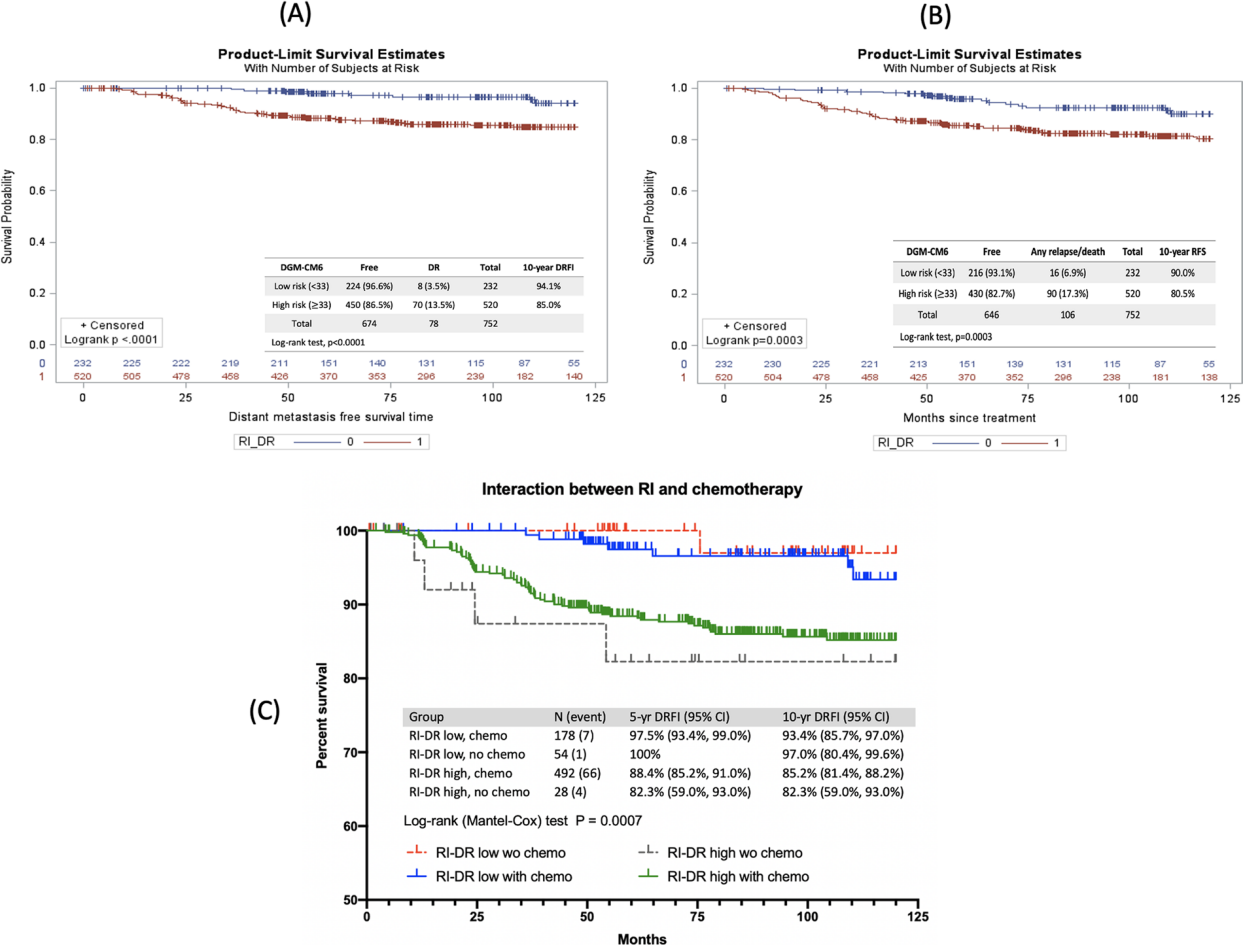
DGM-CM6 and distant recurrence-free interval (DRFI) and relapse-free survival (RFS). (**A**,**B**) DRFI and RFS of low- and high-risk groups divided by DGM-CM6 (RI-DR); X and Y axes of the Kaplan-Meier estimate plots show the follow-up interval (months) and estimated probability of events, respectively. (**C**) The interaction between DGM-CM6 (RI-DR) and adjuvant chemotherapy (DRFI as an event): (1) Blue line: low RI-DR and chemotherapy; (2) Red line: low RI-DR and no chemotherapy; (3) Green line: high RI-DR and chemotherapy; (4) Black line: high RI-DR and no chemotherapy.

We examined the relationship between DGM-CM6 and 10-year DRFI and RFS **(**Fig. 1**)**. The results revealed that 3.5% (8/232) of the low-risk patients had DR and 6.9% (16/232) experienced any type of relapse or death; whereas 13.5% (70/520) of the high-risk group patients had DR and 17.3% (90/520) experienced any type of relapse or death. The estimated 10-year DRFI for low- and high-risk patients by the DGM-CM6 was 94.1% and 85.0% (*P* < 0.0001); and 10-year RFS was 90.0% and 80.5% (*P* = 0.0003), respectively **(**Fig. 1A,B).

Subgroup analyses revealed that DGM-CM6 (RI-DR) and DGM could distinguish the low- and high-risk patients in luminal, HER2, and triple-negative EBC **(**Supplementary Table S6**)**. However, DGM score and RI-DR were not significant factors in patients with HER2-overexpressed and triple-negative breast cancer; the low-risk group had a trend towards a better outcome than the high-risk group. When we confined the analysis to luminal N0-N1 patients, DGM and RI-DR could significantly distinguish the low- and high-risk patients (Supplement Table S6).

For the interaction between DGM-CM6 (RI-DR) and chemotherapy, RI-DR was capable of classifying low- and high-risk N0-2 patients as 10-year DRFI regardless of chemotherapy administration. The 10-year DRFI for low- and high-risk patients who did not receive chemotherapy was 97.0% and 82.3% (*P* = 0.012), respectively. The corresponding rates in patients receiving chemotherapy were 93.4% and 85.2% (*P* = 0.0008), respectively (Fig. 1C). The int eraction between RI-DR and chemotherapy using RFI, DRFS, and RFS as study endpoints was shown in Supplementary Figs. S1–S3.

### Comparison to PAM50 intrinsic subtypes

**Figure 2:**
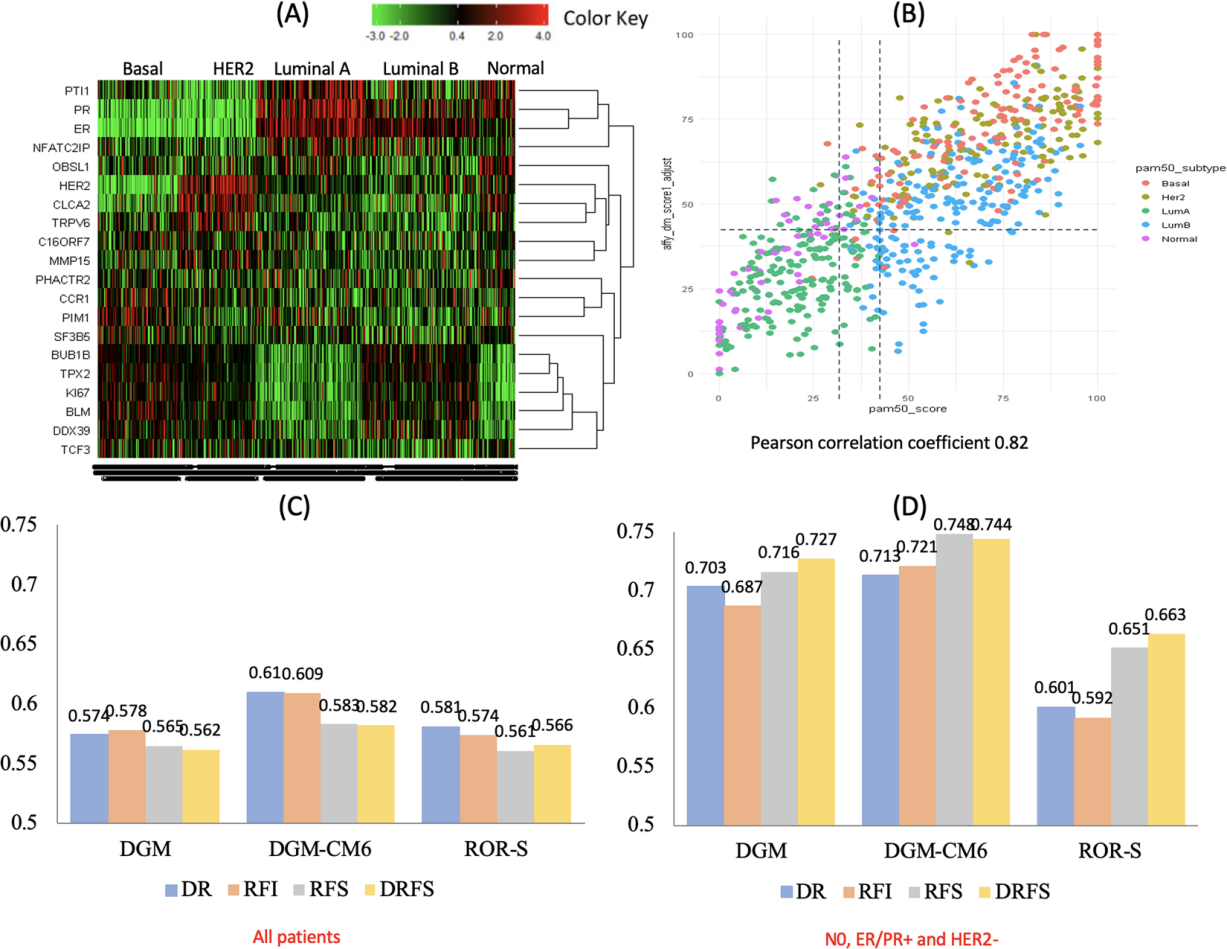
Differentiation of subtypes. (**A**) Heatmap of DGM, ESR1, PgR, HER2, and MKI67 gene expression levels in all 752 patients (X-axis for PAM50 subtypes); Unsupervised clustering DGM genes, ESR1, PgR, HER2 and MKI67 (Y-axis); (**B**) DGM score distribution according to PAM50 subtypes and ROR scores. The X-axis for ROR score; Y-axis for DGM score. PAM50 subtypes: Orange dots represent the basal-like subtype, grey dots the HER2 subtype, green dotes luminal A, blue dots luminal B, and pink dots represent the normal type; (**C**) C-indices for all subtypes; (**D**) C-indices for N0 luminal subtype (ER/PR+ and HER2−) patients only.

According to research-based PAM50 intrinsic subtypes, a heatmap was generated by unsupervised clustering of all 752 patients combining our genomic panel with IHC4 genes (ER, PR, HER2 and MKI67). Our gene panel differentiates each subtype correctly (Fig. 2A); for example, BUB1B, TPX2, BLM, and DDX39 were clustered together with MKI67; furthermore, the panel was capable of distinguishing luminal A from luminal B subtypes. TRPV6 and CLCA2 were clustered together with ERBB2 and the HER2 subtype was differentiated from other subtypes.

The score distributions of DGM were significantly different among PAM50 intrinsic subtypes (Fig. 2B). Luminal A patients had the lowest DGM scores among all subtypes (*P* < 0.0001). PAM50 ROR score (ROR-S) low-risk patients were usually classified as low-risk by DGM; however, DGM further classified some patients in normal-like and luminal B subtypes as low-risk. DGM divided the ROR-S low-risk group into low-risk and high-risk groups; the former had a 10-year DRFI of 93.5% (86.3%, 97.0%) and the latter 77.1% (53.1%, 89.9%) (*P* = 0.0019) (Table 2). DGM also identified low-risk patients in the ROR-S intermediate-risk group with a 10-year DRFI of 95.7% (87.1%, 98.6%). The gene expression levels of DGM-low and -high patients were significantly different (Supplementary Table S4).

**Table 2:** PAM50 ROR score (ROR-S) risk classification and 10-year DRFI by DGM and RI-DR scores.

PAM50 ROR-S	DGM-low 10-year DRFI		DGM-high 10-year DRFI		Log-rank p-value
	Patient #	% (95% CI)	Patient #	% (95% CI)	
Low	169	93.5% (86.3%, 97.0%)	26	77.1% (53.1%, 89.9%)	0.0019
Intermediate	83	95.7% (87.1%, 98.6%)	113	88.8% (81.0%, 93.5%)	0.0831
High	21	64.1% (33.6%, 83.4%)	340	85.2% (80.6%, 88.8%)	0.0883
	**RI-DR low 10-year DRFI**		**RI-DR high 10-year DRFI**		
Low	151	94.6% (86.7%, 97.8%)	44	80.5% (62.9%, 90.3%)	0.0016
Intermediate	66	98.5% (89.6%, 99.8%)	130	88.5% (81.3%, 93.0%)	0.0263
High	15	77.4% (31.5%, 94.5%)	346	84.2% (79.5%, 87.9%)	0.8150

Similarly, DGM combined with clinical variables (DGM-CM6 or RI-DR) separated ROR-S low- and intermediate-risk patients into low- and high-risk groups significantly (Table 2).

### Concordance index (C-index)

Comparing the performance of DGM, DGM-CM6, and PAM50 ROR scores from the validation dataset, the C-index by DGM, DGM-CM6, and ROR for RFS in all patients was 0.565, 0.583, and 0.561, respectively (Fig. 2C). The corresponding C-index in N0, ER+/PR+, and HER2- patients was 0.716, 0.748, and 0.651, respectively (Fig. 2D).

### Uni- and multivariate analyses of the internal validation cohort

Univariate analysis with the Cox regression model revealed that RI-DR was a prognostic factor associated with DRFI, RFI, DRFS, and RFS with hazard ratios of 4.0 (95% CI, 1.9–8.3), 3.8 (1.9–7.6), 2.6 (1.5–4.5) and 2.6 (1.5–4.4), respectively. Tumour stage, nodal status, and tumour grading were also associated with prognosis (all *P* < 0.05). Detailed information is illustrated in Supplementary Table S5.

Multivariate analysis adjusted for age, T or N stage, ER/PR/HER2 status, tumour grade, and LVI by stepwise selection revealed that the RI-DR high-risk group and N2 category were poor prognostic factors for DRFI, RFI, DRFS, and RFS with hazard ratios of 3.8 (1.6–9.0), 3.5 (1.5–8.1), 3.2 (1.6–6.3), and 3.1 (1.6–6.0), respectively (Table 3).

**Table 3:** Multivariate analysis.

Parameter		DRFI		RFI		DRFS		RFS	
		HR (95% CI)	P-value	HR (95% CI)	P-value	HR (95% CI)	P-value	HR (95% CI)	P-value
Age	<40	1.0 (0.5–2.3)	0.9113	1.1 (0.5–2.4)	0.8012	0.8 (0.4–1.5)	0.4521	0.8 (0.4–1.6)	0.5555
	40–60	0.8 (0.4–1.7)	0.625	0.9 (0.5–1.8)	0.8634	0.6 (0.4–1.1)	0.1018	0.7 (0.4–1.2)	0.1961
	>60	Ref		Ref		Ref		Ref	
T stage	T1	Ref		Ref		Ref		Ref	
	T2	1.5 (0.8–2.6)	0.1845	1.6 (0.9–2.7)	0.0986	1.4 (0.9–2.3)	0.1629	1.5 (0.9–2.4)	0.0886
	T3	2.5 (0.8–7.8)	0.108	2.6 (0.8–7.9)	0.0987	2.1 (0.8–5.8)	0.1348	2.2 (0.8–5.8)	0.1295
N stage	N0	Ref		Ref		Ref		Ref	
	N1	1.6 (0.9–3.0)	0.1237	1.5 (0.8–2.8)	0.1659	1.7 (1.0–2.9)	0.0749	1.6 (0.9–2.7)	0.0989
	N2	4.2 (2.1–8.4)	<0.0001	4.1 (2.1–7.9)	<0.0001	3.6 (1.9–6.8)	<0.0001	3.6 (1.9–6.6)	<0.0001
ER/PR status	Both neg.	1.1 (0.6–1.8)	0.8453	1.0 (0.6–1.6)	0.8919	0.7 (0.5–1.2)	0.2329	0.7 (0.5–1.1)	0.16
	ER/PR pos.	Ref		Ref		Ref		Ref	
HER2	Neg.	Ref		Ref		Ref		Ref	
	Pos.	1.0 (0.6–1.7)	0.9571	1.0 (0.6–1.7)	0.8472	0.9 (0.6–1.4)	0.6851	0.9 (0.6–1.5)	0.784
Grade	1	Ref		Ref		Ref		Ref	
	2	1.8 (0.5–6.6)	0.3509	2.2 (0.6–8.0)	0.2149	2.2 (0.8–6.1)	0.1162	2.5 (0.9–6.7)	0.0741
	3	1.1 (0.3–4.1)	0.9413	1.5 (0.4–5.6)	0.5862	1.5 (0.5–4.4)	0.444	1.8 (0.6–5.4)	0.2597
LVI	Absent/focal	Ref		Ref		Ref		Ref	
	Prominent	0.7 (0.4–1.2)	0.1818	0.6 (0.4–1.1)	0.1163	0.7 (0.4–1.1)	0.1331	0.6 (0.4–1.1)	0.0857
Surgery	MRM	0.8 (0.4–1.3)	0.337	0.7 (0.4–1.2)	0.1824	1.0 (0.6–1.6)	0.9911	0.9 (0.6–1.4)	0.6674
	BCT	Ref		Ref		Ref		Ref	
Chemo	No	Ref		Ref		Ref		Ref	
	Yes	0.5 (0.2–1.4)	0.1864	0.4 (0.1–0.9)	0.0259	0.2 (0.1–0.5)	<0.0001	0.2 (0.1–0.4)	<0.0001
DGM-CM6	Low	Ref		Ref		Ref		Ref	
	High	3.8 (1.6–9.0)	0.0028	3.5 (1.5–8.1)	0.0028	3.2 (1.6–6.3)	0.0009	3.1 (1.6–6.0)	0.0009

Multivariate analysis for each subtype revealed that RI-DR was an independent prognostic factor for DRFI, RFI, DRFS, and RFS in luminal subtype **(**Supplementary Table S7**)**. RI-DR in HER2 subtype had hazard ratios of 3.7 (0.4–33.3), 4.6 (0.5–40.3), 1.7 (0.4–7.1), and 2.0 (0.5–7.9) for DRFI, RFI, DRFS, and RFS, respectively **(**Supplementary Table S8**)**. Multivariate analysis for triple-negative subtype could not be performed due to none recurrence being observed in low-risk patients.

### Validation in an external dataset

**Figure 3:**
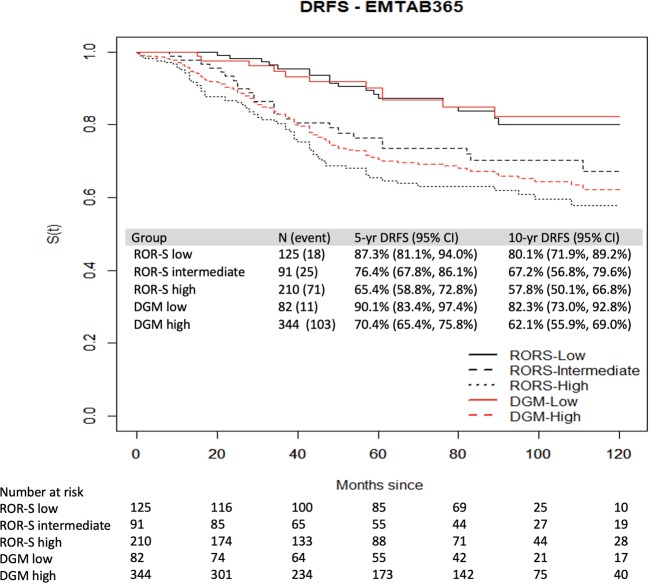
DRFS of patients from the EMTAB-365 dataset. DRFS of low-, intermediate- and high-risk groups divided by PAM50 (black color); and low- and high-risk groups divided by DGM-CM6 (red color).

The performance of DGM (clinical data was inadequate to test DGM-CM6) in predicting the outcomes of N0-2 patients from the EMTAB-365 dataset revealed that the 10-year DRFS was 62.1% in the high-risk group and 82.3% in the low-risk group (*P* < 0.0001) (Fig. 3). According to the PAM50, the ROR-S low-, intermediate- and high-risk patients had 10-year DRFS rates of 80.1%, 67.2% and 57.8%, respectively (Fig. 3).

## Discussion

The new clinical-genomic model DGM-CM6 serves as an independent prognostic factor in patients with N0-N2 primary operable breast cancer, especially the luminal subtype; however, its prognostic value in non-luminal subtype needs to be confirmed with more data. The hazard ratios for DRFI and RFS were 3.8 (1.6–9.0, *P* = 0.0028) and 3.1 (1.6–6.0, *P* = 0.0009), respectively (Table 3**)**. This model also divided PAM50 ROR low- and intermediate-risk patients into different risk groups **(**Table 2**)**. The 10-year rates of DRFI in RI-DR low-risk and ROR low/intermediate-risk groups were excellent, ranging from 94.6% to 98.5%. The data obtained in our study suggest that our model can identify high-risk patients from the ROR low-risk group and low-risk patients from the ROR intermediate-risk group (Table 2). As a result, 44/192 (22.9%) PAM50 luminal A patients were identified as high-risk and 43/212 (20.3%) luminal B patients as low-risk **(**Table 1**)**.

Although the multi-gene panel was initially developed without considering breast cancer subtypes, the heatmap and correlation analyses revealed that our panel can differentiate among PAM50 intrinsic subtypes (Fig. 2A,B). The heatmap showed that the gene expression levels of *BUB1B*, *TPX2*, *BLM and DDX39* are different between PAM50 luminal A and B subtypes. Other researchers have made similar observations; BUB1B is associated with poor prognosis in luminal A breast cancer^14^. TPX2 is the most well-connected gene within a proliferation network; its knockdown significantly affects metastasis but not tumour proliferation in oestrogen receptor-positive tumours^15^. Bloom syndrome helicase (BLM) has key roles in homologous recombination repair; PAM50 luminal A subtype is more likely to express low levels of BLM mRNA^16^.

Concordant statistics using the validation dataset revealed DGM-CM6 had higher C-indices than DGM and PAM50 ROR scores (Fig. 2C). This is understandable as DGM-CM6 incorporates clinical information in the model that might increase the C-index. Confined to node-negative, ER+/PR+ and HER2-negative patients, the C-indices of DGM and DGM-CM6 for DRFS and RFS were 0.72–0.75; however, the C-index of ROR-S was 0.65–0.66 (Fig. 2D). This may be related to the fact that our dataset is based on an Asian population with reduced odds of the basal-like subtype and apparent ethnicity differences^17^. The C-index of ROR-S for post-menopausal node-negative luminal women in anastrozole or tamoxifen alone or combined randomised clinical trials was reported as 0.78^18^.

The main goal of adjuvant chemotherapy is to reduce the risk of distant recurrence. The current study demonstrated very low-risk DR within 5 years in the DGM-CM6 low-risk group. However, some late recurrences developed after 5 years (Fig. 1A). Patients in the current study received hormonal therapy for only 5 years; the DR after 5 years was probably related to the duration of hormonal therapy. The type and risk of recurrence vary significantly among different molecular subtypes; furthermore, our genomic information is highly correlated with the PAM50 subtype. Numerous multi-gene panels or clinical-genomic models have been developed to assist in decision making for adjuvant systemic therapy. However, most of them focus on luminal subtypes and are rarely shown to play a role in basal-like or HER2 positive subtypes. In our gene panel, TRPV6 and CLCA2 were clustered together with ERBB2 and could differentiate HER2 from non-HER2 subtypes **(**Fig. 2A). Both genes are related to ion channel pathway control^19,20^ TRPV6 expression leads to reduction in basal calcium influx and cellular proliferation and is significantly elevated in basal-like and HER2 subtypes^19^. CACL2 is a tumour suppressor, involved in the p53 tumour suppressor network and has a significant effect on cell migration and invasion^21^. These 2 genes could be novel targets for HR-negative breast cancer^19^.

For patients with HER2 positive breast cancer treated with curative surgery, adjuvant trastuzumab for one year is the standard care. However, identifying patients, who are at a higher risk of recurrence and would, therefore, benefit more from novel anti-HER2 agents such as pertuzumab and neratinib is paramount. There is an urgent need for a predictive tool to guide the systemic treatment strategies of these patients. Our clinical-genomic model can classify breast cancer patients into high recurrence risk and low recurrence risk regardless of molecular subtypes, which has the potential to help clinicians make more informed decisions about systemic treatments.

A Korean group has developed a clinical-genomic model (GeneWell BCT), which consists of 6 prognostic genes and 2 clinical risk factors and can divide pN0-N1, ER/PR-positive and HER-2 negative patients into low- and high-risk groups^22^. Comparison of GenesWell BCT score with ODX RS revealed that BCT score classified more low-risk patients than RS in patients aged 50 years or less (73.0% versus 33.6%)^7^. Since Asian breast cancer patients are usually pre-menopausal^23^, further studies, including our model, are necessary to identify which test is more accurate in this subpopulation.

There were some limitations to our study. First, the ideal prognostic validation dataset should recruit only patients who have not received systemic therapies because the risk of recurrence after adjuvant therapy may be underestimated. We had 82 (10.9%) patients who did not receive chemotherapy, but this number was too small for further analysis. It is clear that this study cannot provide adequate information for patients to make a decision about adjuvant chemotherapy. However, the potential prognostic value of our DGM-CM6 model should be noted for the significant difference between the low- and high-risk breast cancer recurrence in large cohorts. Second, most patients with HER2-positive breast cancer did not receive anti-HER2 therapy. The utility of this model in the era of anti-HER2 treatment is unclear. Finally, only a few triple negative breast cancer patients were low-risk according to our model; further investigation is necessary for this group.

In conclusion, we developed a model combining genomic and clinical information as a prognostic tool for non-metastatic breast cancer. This multi-gene model can provide not only clinical outcome information before treatment but also may play a tool to assist in the risk-benefit judgement of systematic adjuvant treatments, especially in Asian patients.

## Materials and Methods

### Patient population

**Figure 4:**
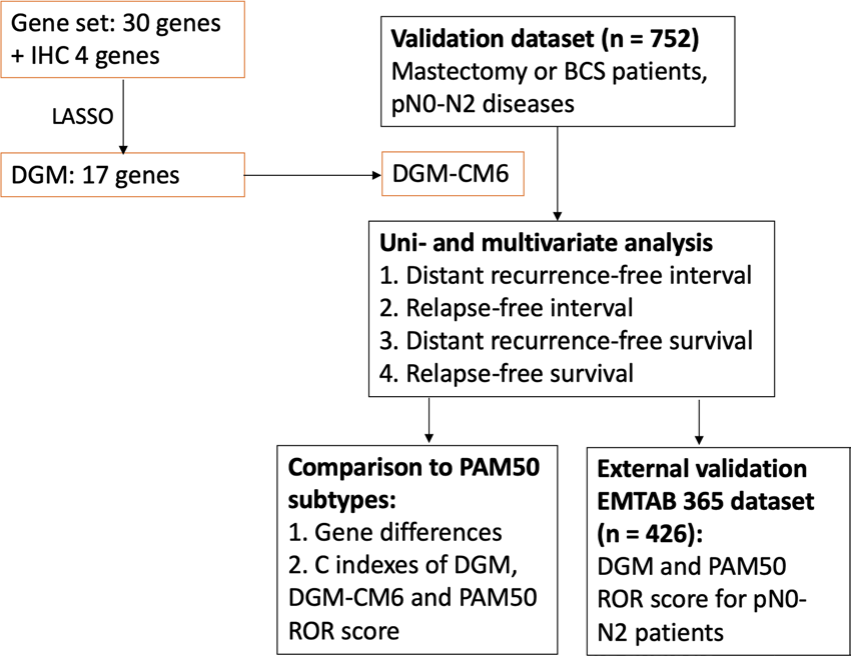
Consolidated Standards of Reporting Trials for this study. Using LASSO to select significant genes. Abbreviation: IHC: immunohistochemistry; DGM: Distant genomic model; CM6: Clinical model 6 (age, lymphovascular invasion, oestrogen receptor, lymph node status, tumor size and grade); ROR: risk of recurrence; LASSO: Least Absolute Shrinkage and Selection Operator.

Breast cancer patients, who had undergone microarray analysis of their primary tumour were enrolled in this study. The Consolidated Standards of Reporting Trials (CONSORT) flow diagram for this study is shown in Fig. 4. Details of the training and testing information for DGM-CM6 has been reported in our previous publication **(**Supplementary Tables S1 and S2**)**^13^. This study focused on validation using a dataset obtained from the Affymetrix platform.

The internal validation cohort consisted of 752 patients, who had a microarray study performed for their primary tumours. This study was performed in a prospective way that all alive participants gave written informed consent to use their frozen tumor tissues from the biobank for the purpose to identify poor or good gene expression profiling. The inclusion criteria were pathology stage pN0-2 (0–9 axillary lymph nodes were positive) breast cancer patients after primary surgery with either mastectomy or breast-conserving surgery (BCS). Patients who had preoperative chemotherapy and pN3, T4, and/or M1 disease were excluded. The protocol and informed consent documents were reviewed and approved by the institutional review board (IRB) of the Koo Foundation Sun Yat-Sen Cancer Center in Taipei, Taiwan (IRB no. 20131001 A).

The EMTAB-365 dataset was used as the external validation cohort, which is the most extensive dataset using Affymetrix U133 Plus 2.0 microarray to analyse gene expression profiles of primary tumour tissues^24^. A total of 426 patients with pN0-N2 regardless of breast subtypes and microarray data were included (http://www.ebi.ac.uk/arrayexpress).

### Affymetrix microarray and PAM50 subtyping

The mRNA microarray results were reported previously^8,25^ RNA was extracted from primary tumour tissue using TRIZOL reagent (Invitrogen/Thermo Fisher Scientific, Waltham, MA, USA) and purified with an RNEASY Mini Kit (Qiagen, Hilden, Germany); the purity was evaluated with an Agilent 2100 Bioanalyzer (Agilent, Santa Clara, CA, USA). According to the Affymetrix protocol, hybridisation targets were prepared from total RNA and hybridised to U133 Plus 2.0 (U133P2) arrays (Affymetrix, Santa Clara, CA, USA). The details of the study protocol were reported previously^25^. Each patient was assigned to an intrinsic molecular subtype of breast cancer (luminal A, luminal B, HER2-enriched, basal-like and normal-like) using the research-based PAM50 subtyping^26,27^.

External validation Affymetrix U133P2 dataset was obtained from ArrayExpress (EMTAB-365). Raw CEL files were pre-processed using the robust multi-array average method in the affy package of R software^28,29^. Quantile normalisation was performed to reduce potential systematic biases. The classification of PAM50 subtypes and calculation of risk of recurrence (ROR) score were performed using genefu R package^26,30,31^.

### Algorithm of DGM and DGM-CM6

The algorithm for the DGM is summarised as follow:

$${\rm{DGM}}\,{\rm{score}}=\beta 1\times {\rm{Gene}}1+\beta 2\times {\rm{Gene}}2+\ldots +{\beta }_{{\rm{N}}}\times {\rm{Gene}}\,{\rm{N}},\,{\rm{N}}\le 17\,({\rm{scores}}\,{\rm{rescaled}}\,{\rm{to}}\,1\mbox{--}100)$$

The RI-DR score was calculated in 2 steps: 1) the genetic score was calculated as described above; and 2) clinical and genetic scores were integrated. The algorithm is summarised as follows:

$${\rm{D}}{\rm{G}}{\rm{M}}-{\rm{C}}{\rm{M}}6\,({\rm{R}}{\rm{I}}-{\rm{D}}{\rm{R}}){\rm{s}}{\rm{c}}{\rm{o}}{\rm{r}}{\rm{e}}={{\rm{H}}}_{1}\times {\rm{D}}{\rm{G}}{\rm{M}}\,{\rm{s}}{\rm{c}}{\rm{o}}{\rm{r}}{\rm{e}}+{{\rm{H}}}_{2}\times {\rm{c}}{\rm{l}}{\rm{i}}{\rm{n}}{\rm{i}}{\rm{c}}{\rm{a}}{\rm{l}}\,{\rm{s}}{\rm{c}}{\rm{o}}{\rm{r}}{\rm{e}}({\rm{C}}{\rm{M}}6)\,({\rm{s}}{\rm{c}}{\rm{o}}{\rm{r}}{\rm{e}}{\rm{s}}\,{\rm{r}}{\rm{e}}{\rm{s}}{\rm{c}}{\rm{a}}{\rm{l}}{\rm{e}}{\rm{d}}\,{\rm{t}}{\rm{o}}\,1-100)$$

### Statistical methods

The Kaplan-Meier method was used to estimate the 10-year relapse-free survival (RFS), DR-free survival (DRFS), and DR-free interval (DRFI); the log-rank test was used to examine whether the difference in survival curves was significant. All statistical analyses were performed using R v.3.4.1 (http://www.R-project.org/) and SAS v.9.4 (SAS Institute). *P* < 0.05 was considered significant.

Patients with DGM score cut-offs $<$ 41 and ≥41 were considered low- and high-risk, respectively. Patients with DGM-CM6 (RI-DR) scores ≥ 33 and <33 were defined as having a high and low-risk of distant metastasis, respectively^13^. Using these predefined cut-offs, we examined the performance of CM6, DGM and RI-DR in training, testing and validation datasets **(**Supplementary Table S9).

### Protocol approval

The Bio-bank Ethics Committee and the IRB of the Koo Foundation Sun Yat-Sen Cancer Center approved this study (approval numbers 20131001A and 20150327A). The committee confirmed that all research was performed in accordance with relevant guidelines/regulations.

## Supplementary information

Supplementary information.
