# Supplementary material for: A new clinical-genomic model to predict 10-year recurrence risk in primary operable breast cancer patients
Source: Sci Rep. 2020 Mar 17;10:4861. doi: 10.1038/s41598-020-61535-9 (PMC7078190; doi:10.1038/s41598-020-61535-9)
Supplement: Supplementary file 1 — Supplementary information. [file 41598_2020_61535_MOESM1_ESM.pdf]

A new clinical-genomic model to predict 10-year recurrence risk in primary operable breast cancer patients

Tzu-Ting Huang^ (1), Lei Lei^ (2,3), Ching-Hsuan Andre Chen (4), Tzu-Pin Lu (4), Chung-Wen, Jen (5), Skye Hung-Chun Cheng (5)*

Departments of *(1) Research and (5) Radiation Oncology, Koo Foundation Sun Yat-Sen Cancer Center, Taipei, Taiwan*

(2) Breast Oncology Department, Zhejiang Cancer Hospital, ZheJiang, China

(3) Visiting Scholar, University of Mississippi Medical Center, MS, USA

(4) Epidemiology and Preventive Medicine, Department of Public Health, National Taiwan University, Taipei, Taiwan

Contents:

1. Supplementary Table S1. Baseline characteristics of subjects in training and testing datasets
2. Supplementary Table S2. Training and testing datasets for clinical-genomic model prediction of distant metastasis
3. Supplementary Table S3. Baseline characteristics in patients with or without chemotherapy
4. Supplementary Table S4. Two-sample t test
5. Supplementary Table S5. Univariate analysis
6. Supplementary Table S6. Subgroup analysis of DRFI according to IHC breast cancer subtypes, DGM, and RI-DR
7. Supplementary Table S7-S8. Multivariate analysis for luminal and HER2
8. Supplementary Table S9. C-index in training, testing and validation datasets
9. Supplementary Fig. S1: Interaction between RI-DR and chemotherapy (RFI)
10. Supplementary Fig. S2: Interaction between RI-DR and chemotherapy (DRFS)
11. Supplementary Fig. S3: Interaction between RI-DR and chemotherapy (RFS)

SUPPLEMENTARY TABLES

Supplementary Table S1. Baseline characteristics of subjects in training and testing datasets

Variable	Training dataset (n = 112)	Testing dataset (n = 46)	*P* value
Median follow-up (months)	74.7	49.6	
Age (years)			0.7977
< 40	14 (12.5%)	7 (15.2%)	
40–60	74 (66.1%)	31 (67.4%)	
> 60	24 (21.4%)	8 (17.4%)	
Surgery			0.0005
Mastectomy	112 (100%)	40 (87.0%)	
Breast-conserving surgery	0 (0%)	6 (13.0%)	
T stage			0.29
T1	55 (49.1%)	17 (37.0%)	
T2	56 (50%)	29 (63.0%)	
T4	1 (0.9%)	0 (0%)	
N stage			0.247
N0	79 (70.5%)	32 (69.6%)	
N1	26 (23.2%)	7 (15.2%)	
N2	3 (2.7%)	3 (6.5%)	
N3	4 (3.6%)	4 (8.7%)	
Estrogen/progesterone receptor status			0.0901
Both negative	33 (29.5%)	20 (43.5%)	
Estrogen or progesterone (+)	79 (70.5%)	26 (56.5%)	
HER2 overexpression			0.1069
Negative	65 (58.0%)	33 (71.7%)	
Positive	47 (42.0%)	13 (28.3%)	
Lymphovascular invasion			0.7879
Absent/focal	93 (83.0%)	39 (84.8%)	
Prominent	19 (17.0%)	7 (15.2%)	
Tumor grade			0.563
Grade I	16 (14.3%)	4 (8.7%)	
Grade II	43 (38.4%)	17 (37.0%)	
Grade III	53 (47.3%)	25 (54.4%)	
Adjuvant chemotherapy			0.0431
No	24 (21.4%)	17 (37.0%)	
Yes	88 (78.6%)	29 (63.0%)	
Adjuvant hormonal therapy			0.043
No	39 (34.8%)	24 (52.2%)	
Yes	73 (65.2%)	22 (47.8%)	
Adjuvant trastuzumab			0.6725
No	107 (95.5%)	45 (97.8%)	
Yes	5 (4.5%)	1 (2.2%)	
Adjuvant radiotherapy			0.2655
No	102 (91.1%)	39 (84.8%)	
Yes	10 (8.9%)	7 (15.2%)	

HER2, human epidermal growth factor receptor 2.

Supplementary Table S2. Training and testing datasets for clinical-genomic model prediction of distant metastasis

	DR prediction by DGM						
**DR risk**	Training dataset			Testing dataset			
	Free (%)	DR (%)	5-year DRFI		Free (%)	DR (%)	5-year DRFI
**Low < 41**	40 (100%)	0 (0%)	100%	Low < 41	23 (88.5%)	3 (11.5%)	81.5%
**High ≥ 41**	46 (63.9%)	26 (36.1%)	67.0%	High ≥ 41	15 (50.0%)	15 (50.0%)	50.1%
**Total**	86	26		Total	28	18	
		$P < 0.0001$				$P = 0.0254$	

	DR prediction by DGM-CM6 (RI-DR)						
**DR risk**	Training dataset			Testing dataset			
	Free (%)	DR (%)	5-year DRFI		Free (%)	DR (%)	5-year DRFI
**Low < 33**	42 (100%)	0 (0%)	100%	Low < 33	15 (83.3%)	3 (16.7%)	81.5%
**High ≥ 33**	44 (62.9%)	26 (37.1%)	66.0%	High ≥ 33	13 (46.4%)	15 (53.6%)	49.3%
**Total**	86	26		Total	28	18	
		$P < 0.0001$				$P = 0.0194$	

DR, distant recurrence; DRFI, distant recurrence-free interval; HER2, human epidermal growth factor receptor 2; LGM-CM4, local genetic model-clinical model 4; LRFI, local/regional recurrence-free interval; LRR, local/regional recurrence; RI-DR, recurrence index for distant recurrence; RI-LR, recurrence index for local recurrence.

Supplementary Table S3. Baseline characteristics in patients with or without chemotherapy

**Variables**	**No C/T (n = 82)**	**C/T (n = 670)**	**P-value**
Median follow-up (Months)	86.9 (0.6-156.3)	96.0 (2.1-169.3)	
Age			0.0133
< 40	6 (7.3%)	115 (17.2%)	
40–60	57 (69.5%)	462 (69.0%)	
> 60	19 (23.2%)	93 (13.9%)	
T stage			< 0.0001
T1	64 (78.1%)	263 (39.3%)	
T2	16 (19.5%)	392 (58.5%)	
T3	2 (2.4%)	15 (2.2%)	
N stage			< 0.0001
N0	75 (91.5%)	289 (43.1%)	
N1	6 (7.3%)	276 (41.2%)	
N2	1 (1.2%)	105 (15.7%)	
ER and PR status			0.04
Both Negative	20 (24.4%)	240 (35.8%)	
ER or PR (+)	62 (75.6%)	430 (64.2%)	
HER2 overexpression			0.0012*
Negative	67 (81.7%)	425 (63.0%)	
Positive	15 (18.3%)	242 (36.1%)	
Indeterminant	0	3 (0.5%)	
Lymphovascular invasion			0.0005
Absent/focal	76 (92.7%)	506 (75.5%)	
Prominent	6 (7.3%)	164 (24.5%)	
Tumor grade			< 0.0001
Grade I	31 (37.8%)	52 (7.8%)	
Grade II	26 (31.7%)	212 (31.6%)	
Grade III	25 (30.5%)	406 (60.6%)	
Adjuvant radiotherapy			< 0.0001
No	47 (57.3%)	128 (19.1%)	
Yes	35 (42.7%)	542 (80.9%)	
Adjuvant H/T			0.2617
No	26 (31.7%)	255 (38.1%)	
Yes	56 (68.3%)	415 (61.9%)	
Adjuvant trastuzumab			0.0002
No	82 (100%)	573 (85.5%)	
Yes	0 (0%)	97 (14.5%)	

Supplementary Table S4. Two-sample t test:

Variable	DGM Low Mean (SD)	DGM High Mean (SD)	DF	T statistics	P value
DDX39	9.87 (0.38)	10.11 (0.40)	389	−5.64	< 0.0001
BUB1B	7.35 (0.80)	8.13 (0.50)	381.85	−11.83	< 0.0001
CCR1	6.88 (0.72)	7.41 (0.86)	244.34	−6.09	< 0.0001
BLM	6.30 (0.43)	6.77 (0.40)	389	−10.59	< 0.0001
C16ORF7	6.82 (0.38)	6.88 (0.40)	389	−1.6	0.111
PIM1	7.78 (0.54)	7.94 (0.53)	389	−2.83	0.0048
TPX2	7.04 (0.81)	7.80 (0.59)	361.05	−10.7	< 0.0001
PTI1	14.56 (0.25)	14.31 (0.20)	344.29	10.6	< 0.0001
TCF3	8.50 (0.41)	8.54 (0.44)	389	−0.93	0.3532
NFATC2IP	9.38 (0.40)	9.13 (0.38)	389	5.98	< 0.0001
OBSL1	8.59 (0.63)	8.44 (0.47)	355.41	2.66	0.0082
MMP15	7.01 (0.42)	7.09 (0.54)	233.46	−1.6	0.1103
CLCA2	6.55 (2.09)	7.47 (2.43)	250.45	−3.75	0.0002
SF3B5	10.79 (0.34)	10.69 (0.33)	389	2.89	0.0041
PHACTR2	6.52 (0.87)	6.67 (1.03)	246.06	−1.47	0.1437
TRPV6	7.25 (0.62)	7.17 (0.69)	389	1.06	0.2891
ER	12.15 (1.27)	10.22 (1.78)	216.94	11.27	< 0.0001
HER2	10.77 (0.75)	11.23 (1.54)	175.36	−3.25	0.0014
PR	10.3 (1.62)	8.63 (1.87)	389	9.22	< 0.0001
KI67	6.18 (0.57)	6.61 (0.45)	341.01	−8.14	< 0.0001

Supplementary Table S5. Univariate analysis (all patients)

		DRFI		RFI		DRFS		RFS	
Parameter		HR (95% CI)	P-value	HR (95% CI)	P-value	HR (95% CI)	P-value	HR (95% CI)	P-value
Age	<40	1.2 (0.6–2.6)	0.5771	1.3 (0.6–2.7)	0.4731	0.9 (0.5–1.7)	0.715	0.9 (0.5–1.7)	0.8292
	40-60	0.8 (0.4–1.5)	0.5461	0.9 (0.5–1.7)	0.7769	0.6 (0.4–1.0)	0.0602	0.7 (0.4–1.1)	0.1182
	>60	Ref		Ref		Ref		Ref	
T stage	T1	Ref		Ref		Ref		Ref	
	T2	2.1 (1.2–3.4)	0.0048	2.0 (1.3–3.3)	0.0042	1.8 (1.1–2.7)	0.0108	1.7 (1.2–2.7)	0.009
	T3	4.0 (1.4–11.6)	0.0116	3.6 (1.2–10.3)	0.0187	3.4 (1.3–8.6)	0.0123	3.1 (1.2–8.0)	0.0183
N stage	N0	Ref		Ref		Ref		Ref	
	N1	1.8 (1.0–3.1)	0.0417	1.5 (0.9–2.6)	0.1056	1.5 (0.9–2.4)	0.093	1.4 (0.9–2.1)	0.1844
	N2	4.5 (2.5–7.9)	< 0.0001	3.9 (2.3–6.7)	< 0.0001	3.3 (2.0–5.5)	< 0.0001	3.1 (1.9–2.1)	< 0.0001
ER/PR status	Both neg.	1.2 (0.7–1.8)	0.534	1.1 (0.7–1.8)	0.5635	0.9 (0.6–1.4)	0.658	0.9 (0.6–1.4)	0.6542
	ER/PR pos.	Ref		Ref		Ref		Ref	
HER2	Neg.	Ref		Ref		Ref		Ref	
	Pos.	1.2 (0.7–1.8)	0.5459	1.2 (0.8–1.9)	0.4121	1.1 (0.7–1.6)	0.8088	1.1 (0.7–1.6)	0.6553
Grade	1	Ref		Ref		Ref		Ref	
	2	3.6 (1.1–11.9)	0.0329	3.8 (1.2–12.3)	0.0283	2.8 (1.1–7.2)	0.0276	2.9 (1.2–7.4)	0.0238
	3	3.0 (0.9–9.7)	0.0635	3.4 (1.0–10.8)	0.0413	2.3 (0.9–5.7)	0.0791	2.5 (1.0–6.2)	0.0514
LVI	Absent/focal	Ref		Ref		Ref		Ref	
	Prominent	1.4 (0.8–2.2)	0.2163	1.2 (0.8–2.0)	0.3781	1.2 (0.8–1.9)	0.3667	1.1 (0.7–1.8)	0.5484
Surgery	MRM	1.2 (0.8–2.0)	0.3719	1.2 (0.7–1.8)	0.544	1.5 (0.9–2.3)	0.0877	1.4 (0.9–2.1)	0.1537
	BCT	Ref		Ref		Ref		Ref	
Chemo	No	Ref		Ref		Ref		Ref	
	Yes	1.6 (0.7–4.0)	0.3024	1.2 (0.6–2.6)	0.6411	0.7 (0.4–1.2)	0.2175	0.6 (0.4–1.1)	0.0936
DGM-CM6	Low	Ref		Ref		Ref		Ref	
	High	4.0 (1.9–8.3)	0.0002	3.8 (1.9–7.6)	0.0001	2.6 (1.5–4.5)	0.0006	2.6 (1.5–4.4)	0.0005

Supplementary Table S6. Subgroup analysis of DRFI according to IHC breast cancer subtypes, DGM, and RI-DR

All patients	Patient number	DR events	5-year DRFI (%)	10-year DRFI (%)	P value
**RI-DR risk group**					
Luminal patients					<0.0001
Low risk	190	7	98.3	93.7	
High risk	148	26	85.2	79.0	
HER2 patients					0.1334
Low risk	34	1	96.2	96.2	
High risk	223	27	88.4	87.2	
TNBC patients					0.3579
Low risk	8	0	100.0	100.0	
High risk	146	15	91.7	89.1	

**DGM risk group**					
Luminal patients					0.0002
Low risk	219	12	96.9	91.8	
High risk	119	21	84.5	79.0	
HER2 patients					0.4973
Low risk	41	3	91.7	91.7	
High risk	216	25	88.9	87.7	
TNBC patients					0.2822
Low risk	11	0	100.0	100.0	
High risk	146	15	91.5	88.9	
**Confined to luminal N0-N1 patients.**					
**RI-DR risk group**					
Luminal patients					0.0121
Low risk	178	7	98.2	93.5	
High risk	113	13	90.1	86.2	
**DGM risk group**					
Luminal patients					0.0234
Low risk	195	9	97.5	92.6	
High risk	96	11	89.8	86.6	

Supplementary Table S7. Multivariate analysis for IHC-based luminal subtype

		**DRFI**		**RFI**		**DRFS**		**RFS**	
Parameter		HR (95% CI)	P-value	HR (95% CI)	P-value	HR (95% CI)	P-value	HR (95% CI)	P-value
Age	< 40	2.4 (0.5–12.0)	0.27	2.7 (0.6–12.9)	0.216	0.7 (0.3–2.0)	0.5578	0.8 (0.3–2.2)	0.6815
	40-60	1.6 (0.4–6.9)	0.5486	1.7 (0.4–7.3)	0.4904	0.6 (0.2–1.3)	0.1952	0.6 (0.3–1.4)	0.2332
	>60	Ref		Ref		Ref		Ref	
T stage	T1	Ref		Ref		Ref		Ref	
	T2	1.8 (0.7–4.6)	0.2194	2.1 (0.8–5.2)	0.1201	1.7 (0.8–3.6)	0.1676	1.9 (0.9–3.9)	0.0959
	T3	4.1 (0.9–18.3)	0.0666	3.9 (0.9–17.5)	0.0724	2.8 (0.7–11.1)	0.1366	2.8 (0.7–11.0)	0.141
N stage	N0	Ref		Ref		Ref		Ref	
	N1	1.6 (0.9–4.4)	0.3551	1.2 (0.5–2.9)	0.7582	1.8 (0.8–4.0)	0.1785	1.4 (0.6–3.0)	0.4134
	N2	4.8 (1.6-13.9)	0.0041	3.4 (1.3-9.2)	0.0143	3.2 (1.3-8.3)	0.0145	2.6 (1.0–6.2)	0.0402
Grade	1	Ref		Ref		Ref		Ref	
	2	1.7 (0.4–6.6)	0.4522	1.9 (0.5–7.1)	0.3589	2.0 (0.7–5.5)	0.2079	2.0 (0.7–5.6)	0.185
	3	1.0 (0.2–4.7)	0.973	1.2 (0.3–5.5)	0.8252	1.3 (0.4–4.3)	0.6408	1.4 (0.5–4.6)	0.5359
LVI	Absent/focal	Ref		Ref		Ref		Ref	
	Prominent	0.3 (0.1–0.8)	0.0134	0.3 (0.1–0.8)	0.017	0.4 (0.2–0.9)	0.0176	0.4 (0.2–0.8)	0.0151
Surgery	MRM	1.0 (0.4–2.2)	0.9296	1.1 (0.5–2.5)	0.8421	1.6 (0.7–3.3)	0.2538	1.7 (0.8–3.6)	0.1766
	BCT	Ref		Ref		Ref		Ref	
Chemo	No	Ref		Ref		Ref		Ref	
	Yes	0.6 (0.1–3.2)	0.5387	0.7 (0.1–3.9)	0.7256	0.2 (0.1–0.6)	0.003	0.3 (0.1–0.7)	0.0078
DGM-CM6	Low	Ref		Ref		Ref		Ref	
	High	3.4 (1.2–9.7)	0.0211	2.9 (1.1–7.8)	0.0345	4.2 (1.8–9.6)	0.0009	3.7 (1.6–8.3)	0.0017

Supplementary Table S8. Multivariate analysis for IHC-based HER2 subtype

		DRFI		RFI		DRFS		RFS	
Parameter		HR (95% CI)	P-value	HR (95% CI)	P-value	HR (95% CI)	P-value	HR (95% CI)	P-value
Age	< 40	0.8 (0.3–2.6)	0.7207	0.8 (0.2–2.5)	0.6768	1.0 (0.4–3.0)	0.9679	1.0 (0.3–2.9)	0.9994
	40-60	0.5 (0.2–1.5)	0.2237	0.7 (0.2–1.8)	0.4341	0.6 (0.2–1.5)	0.2923	0.7 (0.3–1.9)	0.5167
	>60	Ref		Ref		Ref		Ref	
T stage	T1	Ref		Ref		Ref		Ref	
	T2	2.7 (0.9–8.2)	0.0695	2.4 (0.9–6.3)	0.0889	2.1 (0.8–5.1)	0.1179	1.9 (0.8–4.4)	0.1487
	T3	3.0 (0.3–30.4)	0.347	2.4 (0.3–22.5)	0.451	1.9 (0.2–17.6)	0.5736	1.6 (0.2–14.0)	0.6881
N stage	N0	Ref		Ref		Ref		Ref	
	N1	1.1 (0.4–3.4)	0.8193	1.4 (0.5–4.0)	0.5036	1.1 (0.4–2.8)	0.9214	1.3 (0.5–3.2)	0.6295
	N2	5.4 (1.6-18.6)	0.0071	8.5 (2.5-28.7)	0.0006	5.2 (1.7-15.7)	0.0032	7.3 (2.5–21.5)	0.0003
ER/PR status	Both neg.	1.8 (0.8-4.0)	0.1499	1.6 (0.7-3.3)	0.2505	1.4 (0.7-2.8)	0.4144	1.2 (0.6-2.5)	0.5511
	ER/PR pos.	Ref		Ref		Ref		Ref	
Grade	1-2	Ref		Ref		Ref		Ref	
	3	0.5 (0.2–1.5)	0.214	0.6 (0.2–1.7)	0.357	0.6 (0.2–1.4)	0.2107	0.6 (0.3–1.6)	0.3333
LVI	Absent/focal	Ref		Ref		Ref		Ref	
	Prominent	1.0 (0.4–2.5)	0.9743	0.9 (0.4–2.2)	0.017	0.8 (0.3–1.9)	0.6099	0.8 (0.3–1.7)	0.5134
Surgery	MRM	0.3 (0.1–0.7)	0.0113	0.2 (0.1–0.5)	0.001	0.3 (0.1–0.8)	0.016	0.2 (0.1-0.6)	0.0019
	BCT	Ref		Ref		Ref		Ref	
Chemo	No	Ref		Ref		Ref		Ref	
	Yes	0.2 (0.0–1.0)	0.0535	0.1 (0.0–0.5)	0.0043	0.2 (0.0–0.6)	0.009	0.1 (0.0–0.4)	0.0009
DGM-CM6	Low	Ref		Ref		Ref		Ref	
	High	3.7 (0.4–33.3)	0.238	4.6 (0.5–40.3)	0.1723	1.7 (0.4–7.1)	0.4332	2.0 (0.5–7.9)	0.3408

Supplementary Table S9. C-index in training (n = 112), testing (n = 46) and validation datasets (n = 752)

	**CM6**	**DGM**	**DGM-CM6**
Accuracy in training dataset	41.1%	58.9%	60.7%
Sensitivity	100.0%	100.0%	100.0%
Specificity	23.3%	46.5%	48.8%
PPV	28.3%	36.1%	37.1%
NPV	100.0%	100.0%	100.0%
C-index	86%	91%	93%
**Accuracy in testing dataset**	**56.5%**	**60.9%**	**65.2%**
**Accuracy in validation dataset**	**20.5%**	**42.4%**	**39.1%**

Supplementary Fig. S1: Interaction between RI-DR and chemotherapy (RFI)

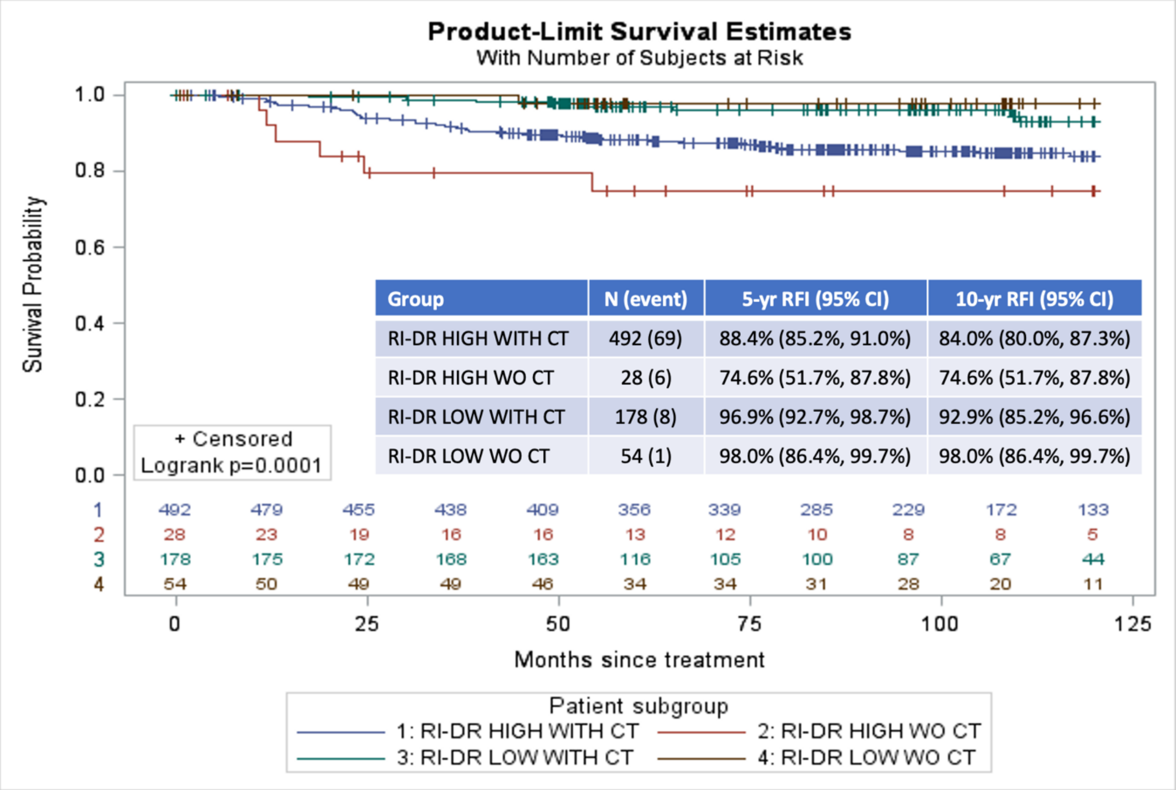

For patients having adjuvant chemotherapy, the 5-year RFI in the low- and high-risk group was significantly different, the 10-year RFI between these two groups was not significantly different. For patients without adjuvant chemotherapy, a trend toward better 5- and 10-year RFI was observed in the RI-DR low-risk group (hazard ratios overlapped in both groups).

Supplementary Fig. S2: Interaction between RI-DR and chemotherapy (DRFS)

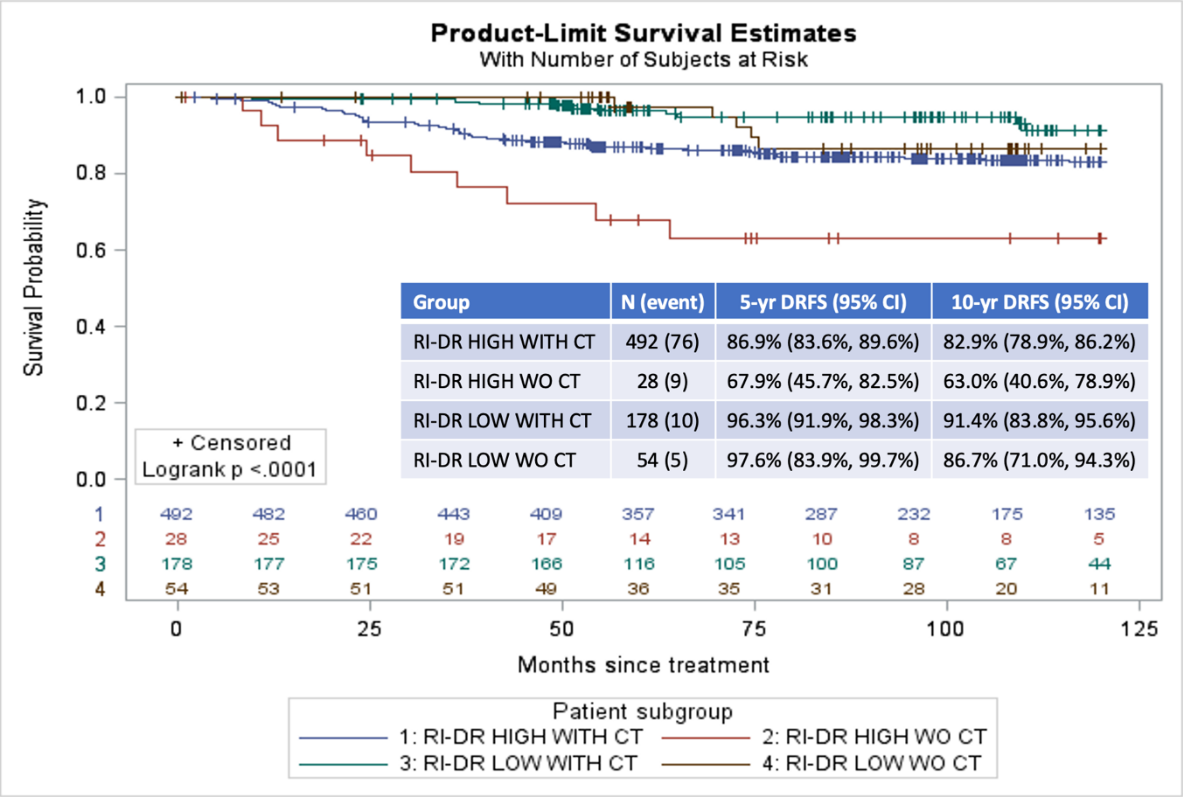

For patients having adjuvant chemotherapy, the 5-year DRFS in the low- and high-risk group was significantly different, however, the 10-year DRFS in both groups was not significantly different. Similar observations were found in patients without adjuvant chemotherapy.

Supplementary Fig. S3: Interaction between RI-DR and chemotherapy (RFS)

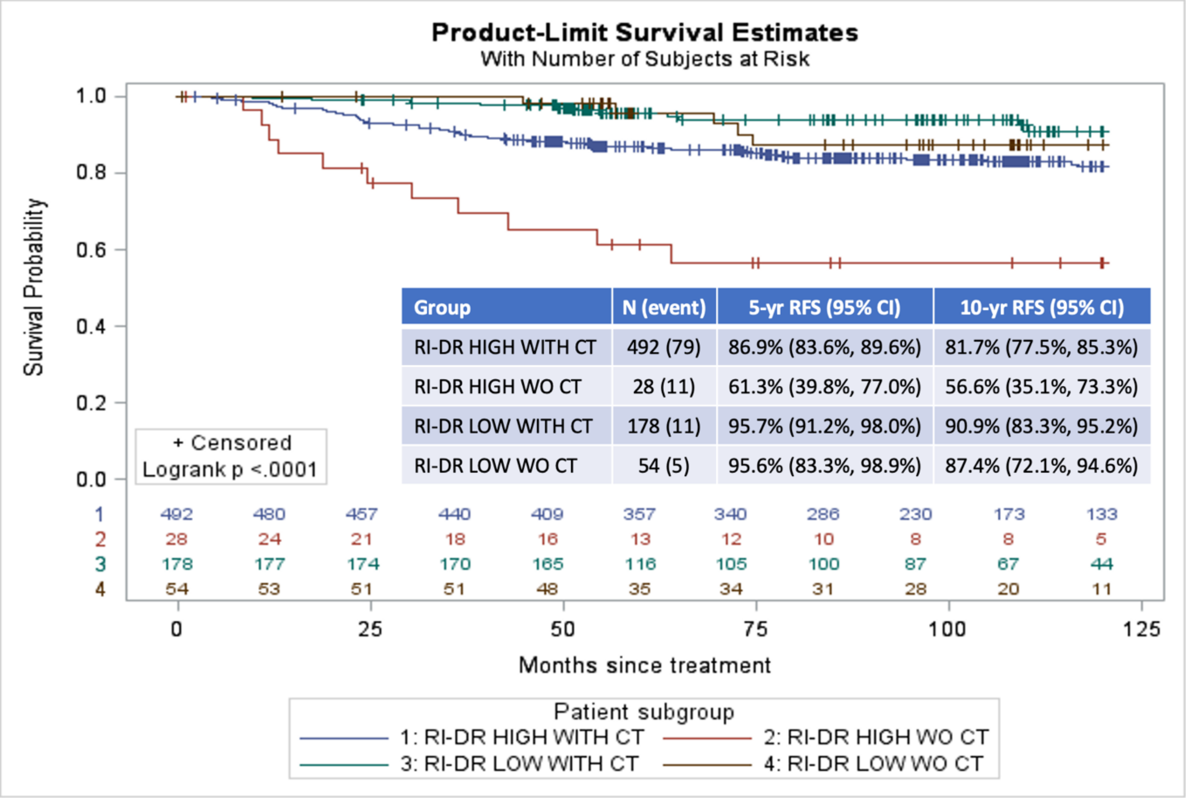

The interaction between chemotherapy and RI-DR using RFS as study endpoint, the observation was similar to DRFS.
